# Dyscalculia and dyslexia in Chinese children with idiopathic epilepsy: Different patterns of prevalence, comorbidity, and gender differences

**DOI:** 10.1002/epi4.12577

**Published:** 2022-01-27

**Authors:** Dazhi Cheng, Xinyang Miao, Haiyan Wu, Chuansheng Chen, Qian Chen, Xinlin Zhou

**Affiliations:** ^1^ 36776 Department of Pediatric Neurology Capital Institute of Pediatrics Beijing China; ^2^ 47836 State Key Laboratory of Cognitive Neuroscience and Learning IDG/McGovern Institute for Brain Research Beijing Normal University Beijing China; ^3^ 47836 Lab for Educational Neuroscience Center for Educational Science and Technology Beijing Normal University Beijing China; ^4^ Institute of Basic Medicine Chinese Academy of Medical Sciences & School of Basic Medicine Peking Union Medical College Beijing China; ^5^ 59193 Centre for Cognitive and Brain Sciences and Department of Psychology University of Macau Taipa Macau; ^6^ Department of Psychological Science University of California Irvine California USA

**Keywords:** dyscalculia, dyslexia, idiopathic epilepsy, prevalence, school children

## Abstract

**Objective:**

The present study aimed to examine the prevalence of dyscalculia, dyslexia, and their comorbidity rates in a large population‐based sample of children with idiopathic epilepsy (N = 2282) and a comparison sample of typically developing schoolchildren (N = 2371).

**Methods:**

Both groups of children were screened using an arithmetic fluency test for dyscalculia and a reading fluency test for dyslexia. Their comorbidity rates were assessed. The prevalence rates of dyscalculia, dyslexia, comorbidity, and isolated dyscalculia/dyslexia (ie, participants with comorbid dyslexia and dyscalculia were excluded) were analyzed.

**Results:**

In both −1.5 SD and −1 SD cutoff criterion, the prevalence rates were about two times higher in children with idiopathic epilepsy than in other schoolchildren; the prevalence rates of isolated dyslexia were higher in children with idiopathic epilepsy than in other schoolchildren (−1 SD: 10.9% vs 8.6%; −1.5 SD: 6.5% vs 4.7%). Meanwhile, comorbidity rates of dyscalculia and dyslexia were higher in children with idiopathic epilepsy than in other schoolchildren (32.7% vs 26.6%; 38.3% vs 23.5%, respectively). Overall, patterns of prevalence rates were different for children with idiopathic epilepsy and schoolchildren, in which children with idiopathic epilepsy had a higher prevalence rate of dyscalculia than dyslexia, while schoolchildren had a higher prevalence of dyslexia than dyscalculia, regardless of cutoff criteria. Interestingly, gender differences in the prevalence rates of all types of learning disabilities were found in schoolchildren, but there were only gender differences in the prevalence rates of dyslexia in children with idiopathic epilepsy.

**Significance:**

The results highlight the vulnerability of children with idiopathic epilepsy for learning disabilities and a differential pattern of gender differences in dyslexia. Moreover, different patterns of prevalence rates suggest that children with idiopathic epilepsy and schoolchildren are more prone to different types of learning disabilities. The findings suggest needs for special interventions of learning disabilities for children with idiopathic epilepsy.

**Key Points**

- School‐age children with epilepsy are at a high risk of developing learning disabilities (mainly including dyscalculia and dyslexia), but so far most studies are of small scale. Even less is known about the prevalence of learning disabilities and their comorbidity rates among children with idiopathic epilepsy in China.
- Chinese children with idiopathic epilepsy had higher prevalence and comorbidity rates of learning disabilities than other schoolchildren.
- Typical developing schoolchildren and children with idiopathic epilepsy had different patterns of prevalence rates for different types of learning disabilities.
- There were significant gender differences in the prevalence of learning disabilities among typical developing schoolchildren, and significant gender differences in the prevalence of dyslexia among children with idiopathic epilepsy.

## 1 INTRODUCTION

Two main types of learning disabilities (LDs) are arithmetic disability (eg, dyscalculia) and reading disability (eg, dyslexia), which afflict about 5% and 6% of school‐age children, respectively.^1^ Children with epilepsy are at a much higher risk of suffering from cognitive impairments and educational underachievement.^2^, ^3^, ^4^, ^5^, ^6^, ^7^ For example, a study on 977 children with epilepsy reported a 56% prevalence rate of learning disabilities.^8^ Another study with a sample of 31 patients diagnosed with BECTS showed that dyslexia occurred in 19.4% of these patients.^9^ Under low achievement model, a study on 164 children with epilepsy found that 20.1% of children scored −1.5 SD below the average for reading and 26.8% for math.^10^

The prevalence rates of learning disabilities also differ by gender. Population‐based studies in Western countries have consistently found boys to be more vulnerable to dyslexia than girls, even after controlling for attention, activity levels, and race.^11^ Similarly, studies of Chinese children have shown that boys were more likely than girls to experience reading difficulty.^12^ However, the results on gender differences in math fluency are mixed. Some studies showed no significant gender differences,^13^, ^14^, ^15^ whereas others found a higher rate of arithmetic disorder among females than among males^16^ or the opposite.^17^, ^18^ Among children with epilepsy, most studies found no significant gender effect,^19^, ^20^, ^21^ but the study by Austin and colleagues^19^ found that male sex was a significant predictor of poor academic achievement in composite, reading, language, and vocabulary for children with epilepsy.

Although previous studies have documented the vulnerability of children with epilepsy for learning disabilities based on relatively small sample sizes (less than 1000 participants), empirical research to examine the prevalence of learning disabilities in a large population‐based sample of Chinese children with epilepsy is still scarce. In addition, the rates of developing learning disabilities in a new domain were four to five times higher in people who already had learning disability in one domain compared to those who never experienced pronounced problems in an academic domain.^22^ For the first time, this study examines the prevalence of learning disabilities, their comorbidity, and gender differences in a large population‐based sample of Chinese children with idiopathic epilepsy (N = 2282) in comparison with a group of typically developing schoolchildren (N = 2371). Since epilepsy increases children's risk to develop learning disabilities, we expect higher prevalence and comorbidity rates of learning disabilities in children with idiopathic epilepsy when compared to other schoolchildren.

## 2 METHODS

### 2.1 Participants

Children with idiopathic epilepsy, a common type of childhood epilepsy, were selected. Idiopathic epilepsy in this study consists of the following conditions: idiopathic generalized epilepsies including childhood absence epilepsy, juvenile absence epilepsy, juvenile myoclonic epilepsy, and generalized tonic–clonic seizures; self‐limited focal epilepsies including benign childhood epilepsy with centrotemporal spikes (BECTS), Panayiotopoulos and Gastaut syndrome; and other frontal lobe, temporal, and parietal lobe epilepsies with unknown etiologies but good response to the antiepileptic drugs.^23^ Approval for this project was granted by the Human Research Ethics Committee at the Capital Institute of Pediatrics (approval no.: SHERLL 2019001). Informed and written consent was obtained from parents or guardians of all participants. For this study, idiopathic epilepsy was diagnosed according to the criteria set by the International League Against Epilepsy.^23^, ^24^ The diagnoses were based on classical clinical features including the onset age, seizure types, and interictal discharges recorded by video‐electroencephalogram (VEEG) monitor, ictal EEG evolution, and imaging features.^23^ BECTS patients were excluded if they had abnormal magnetic resonance imaging (MRI) and a nonrapid eye movement (NREM) sleep discharge index ≥50%. No epilepsy‐related structural abnormalities were present in these children. Children with idiopathic epilepsy comorbid with mental retardation (eg, autism) were excluded because they could not understand the instructions of our screening tests. Subsequently, 2282 children with idiopathic epilepsy were retrospectively enrolled from the Department of Pediatric Neurology at the Capital Institute of Pediatrics (Table 1).

**TABLE 1:** Demographic characteristics of the study participants

Variables	Children with idiopathic epilepsy	Other schoolchildren	*p* Value
Number of participants	N = 2282	N = 2371	N/A
Age (years), mean (SD), range	9.21 (2.34), 6‐17	10.20 (1.58), 6‐16	0.000
Gender (Male/Female)	1446/836	1256/1115	0.000

Abbreviation: N/A, Not applicable.

Other schoolchildren were retrospectively enrolled from a dataset collected by the State Key Laboratory of Cognitive Neuroscience and Learning at Beijing Normal University. The dataset included 2371 children from 73 classes of 9 primary schools in Beijing, Shijiazhuang, China. The classes were randomly selected from these schools. All students from all selected classes participated in the study. Each class included approximately 20‐40 children. The current study's design was approved by the State Key Laboratory of Cognitive Neuroscience and Learning at Beijing Normal University and the school principles. All participants were native Chinese speakers with normal or corrected‐to‐normal vision. Given the fact that autistic children are not able to enroll in regular schools in China, participants in schoolchildren group exclude the children with autism. Participants’ parents or legal guardians provided written informed consent.

According to the definition and screening criteria of dyscalculia and dyslexia,^25^, ^26^, ^27^ children with DD were defined as having scores lower than −1 SD or −1.50 SD in arithmetic or reading performance but above the 25th percentile (−0.67 SD) in Raven's Progressive Matrices. The cutoff criteria (−1 and −1.5 SD) mean that the test performance in children with learning disabilities was lower than −1 or −1.5 SDs from the mean standard scores. For the −1 SD cutoff criterion, the definition of dyscalculia or dyslexia was a score below the 16th percentile for arithmetic fluency or reading fluency. When the more stringent cutoff criterion (ie, −1.5 SD) was applied, the definition of dyscalculia or dyslexia was a score below the 7th percentile for arithmetic fluency or reading fluency. So far, most relevant studies have used −1 SD or both −1 SD and −1.5 SD as the cutoff criteria for learning disabilities.^22^, ^25^, ^26^, ^28^, ^29^, ^30^ Therefore, the definitions of dyscalculia and dyslexia in the current study were aligned with such standards.

Dyslexia was defined using standard scores on reading fluency tests. The criteria for dyslexia include (1) a score equal to or less than the 16th and 7th percentile (−1 and −1.50 SDs from the mean, respectively) for reading fluency; and (2) a score above the 7th percentile (−1.50 SDs from the mean) for general intelligence.

Dyscalculia was defined using standard scores on arithmetic fluency tests. The criteria for dyscalculia include (1) a score equal to or less than the 16th and 7th percentile (−1 and −1.50 SDs from the mean) for arithmetic fluency; (2) a score above the 7th percentile (−1.50 SDs from the mean) for general intelligence.

Comorbid dyslexia‐dyscalculia was defined using standard scores on both reading and math fluency tests. The criteria for comorbid dyslexia and dyscalculia include the following: (1) a score equals to or less than the 16th and 7th percentile (−1 and −1.50 SDs from the mean, respectively) for arithmetic fluency and reading fluency; and (2) a score above the 7th percentile (−1.50 SD from the mean) for reasoning or general intelligence.

Isolated dyslexia was defined using standard scores on reading fluency tests. The criteria for dyslexia include (1) a score equal to or less than the 16th and 7th percentiles (−1 and −1.50 SDs from the mean, respectively) for reading fluency; (2) a score above the 7th percentile (−1.50 SD from the mean) for general intelligence; and (3) a score above the 16th and 7th percentiles (−1 and −1.50 SDs from the mean, respectively) for arithmetic fluency. Participants with comorbid dyslexia and dyscalculia were excluded.

Isolated dyscalculia was defined using standard scores on arithmetic fluency tests. The criteria for dyscalculia include (1) a score equal to or less than the 16th and 7th percentiles (−1 and −1.50 SDs from the mean) for arithmetic fluency; (2) a score above the 7th percentile (−1.50 SDs from the mean) for general intelligence; and (3) a score above 16th and 7th percentiles (−1 and −1.50 SDs from the mean, respectively) for reading. Participants with comorbid dyslexia and dyscalculia were excluded.

### 2.2 Screening tests

#### 2.2.1 Reading fluency

Similar to a previously described task,^31^ a sentence completion task was used to evaluate reading fluency. Items in the task were adapted from textbooks used in schools from the 1st grade to the 12th grade. For each trial, an incomplete sentence was presented in the middle of a computer screen. Participants were instructed to complete the sentence by selecting one of the two possible words presented beneath the sentence by pressing a left key (“Q”) or a right key (“P”). The question remained on the screen until participants responded. There were 120 questions. This was a timed task of 5 minutes.

#### 2.2.2 Arithmetic fluency

Simple subtraction problems were used to measure arithmetic fluency. Participants were not allowed to use paper and pencil. For all 92 simple subtraction problems in the task, the minuends were 18 or smaller and the differences were single‐digit numbers. Two possible answers were presented beneath each problem. Participants were instructed to select the correct answer by pressing the “Q” key to choose the answer on the left and “P” to choose the answer on the right. Each incorrect answer was within ±3 values of the correct answer. This was a timed task of 2 minutes.

#### 2.2.3 Nonverbal matrices reasoning

Raven's Progressive Matrices test was used to assess reasoning or general intelligence.^32^ This task is the simplified version of Raven's Progressive Matrices test. In each trial, figures with a missing segment appeared on the top of the screen. Participants were required to find the rules underlying the presented figure and to select the missing segment of the figure from two choices by pressing the “Q” key to choose the answer on the left and “P” to choose the answer on the right. The test included 80 trials and was a timed task of 3 minutes.

### 2.3 Procedure

All participants in the study received and completed the same three tests: a reading fluency test, an arithmetic fluency test, and a nonverbal matrices reasoning test. Each participant completed the computerized test battery in an examination room. All test procedures were presented on a computer screen, and instructions were given orally. For each test, instructions were given first, followed by a practice session. Participants were allowed to ask the experimenters questions during practice sessions. After the children finished the practice session and resolved their doubts, they could press the space key to begin the formal test. The tasks were administered in the same order for all participants. Each participant was monitored by one tester who was trained in the standardized testing procedures. Participants’ responses were automatically recorded and conveyed over the Internet to a server located in the Key Laboratory for storage. All data were collected between December 2013 and June 2014.

### 2.4 Data analysis

For all tasks, corrected scores were calculated by subtracting the number of incorrect responses from the number of correct responses.^33^ Subsequently, standard z‐scores were calculated for each participant as the corrected score minuses the mean score for a given age year and then divided by the standard deviation for that specific age year.^26^ In order to control for the standards, standard deviations and means of schoolchildren's results were used to calculate z‐scores for children with epilepsy.

An independent Chi‐square test was performed to examine the relation between learning disabilities (dyscalculia/dyslexia/isolated dyscalculia/isolated dyslexia) and each group (children with epilepsy vs other schoolchildren). Participants were then separated into two secondary groups (children with learning disabilities vs children without learning disabilities) to perform a cross‐analysis between epileptic children and school children. Different cutoff criteria (−1 and −1.5 SDs) were used to define learning disabilities. Chi‐square tests were also used to examine gender differences in the prevalence of dyscalculia, dyslexia, isolated dyscalculia, and isolated dyslexia in children with idiopathic epilepsy and other schoolchildren.

Comorbidity rates were calculated for each age group by dividing the number of individuals with dyslexia or dyscalculia by the number of individuals who were classified as having both dyslexia and dyscalculia, which can be captured by this formula below:

$$\mathrm{Comorbidity}\;\mathrm{Rates}=\frac{\mathrm{comorbidity}\;\mathrm{dyslexia}\;\mathrm{and}\;\mathrm{dyscalculia}}{\mathrm{dyslexia}\;\mathrm{and}\;\mathrm{dyscalculia}}$$

## 3 RESULTS

### 3.1 Prevalence

Chi‐square analyses under two standard cutoff criteria of −1 and −1.5 SDs are presented in Table 2. The results show that there were significant differences between groups regarding the prevalence of dyscalculia, dyslexia, isolated dyscalculia, and isolated dyslexia, regardless of the cutoff criterion. Specifically, under both −1 and −1.5 SDs cutoff criteria, children with idiopathic epilepsy had a higher prevalence rate of dyscalculia (−1 SD: 23.2% vs 10.4%, −1.5 SD: 12.3% vs 5.4%, all *P*s < 0.001), dyslexia (−1 SD: 20.8% vs 12.4%, −1.5 SD: 10.5% vs 6.1%, all *P*s < 0.001), isolated dyscalculia (−1 SD: 13.3% vs 6.6%, −1.5 SD: 8.3% vs 4.0%, all *P*s < 0.001), and isolated dyslexia (−1 SD: 10.9% vs 8.6%, −1.5 SD: 6.5% vs 4.7%, all *P*s < 0.05), compared to other schoolchildren. For all learning disabilities except for isolated dyslexia, the prevalence rates were about two times higher in children with epilepsy than in other schoolchildren.

**TABLE 2:** Prevalence of dyscalculia and dyslexia in children with idiopathic epilepsy and other schoolchildren in China under different cutoff criteria (−1 and −1.5 SD)

Groups	Children with idiopathic epilepsy (N = 2282)	Other schoolchildren	χ²
		(N = 2371)	
	% (N)	% (N)	
‐1 SD			
Dyscalculia	23.2 (530)	10.4 (247)	137.125***
Dyslexia	20.8 (474)	12.4 (295)	58.477***
Dyscalculia (Isolated)	13.3 (304)	6.6 (157)	58.479***
Dyslexia (Isolated)	10.9 (248)	8.6 (205)	6.530*
‐1.5			
Dyscalculia	12.3 (281)	5.4 (128)	69.357***
Dyslexia	10.5 (240)	6.1 (145)	29.683***
Dyscalculia (Isolated)	8.3 (189)	4.0 (94)	37.949***
Dyslexia (Isolated)	6.5 (148)	4.7 (111)	7.199**

*P < 0.05, **P < 0.01, ***P < 0.001.

The comorbidity rates of dyscalculia or dyslexia are presented in Tables 3. For children with idiopathic epilepsy, 42.6% (−1 SD) and 32.7% (−1.5 SD) of those with dyscalculia had reading disabilities; 47.6% (−1 SD) and 38.3% (−1.5 SD) of those with dyslexia had arithmetic disabilities. For typically developing schoolchildren, 36.4% (−1 SD) and 26.6% (−1.5 SD) of those with dyslexia had arithmetic disabilities; 30.5% (−1 SD) and 23.5% (−1.5 SD) of those with dyslexia had arithmetic disabilities. In general, the comorbidity rates were relatively high for both children with epilepsy and typically developing schoolchildren, while the former showed noticeably higher comorbidity rates than the latter for both dyscalculia and dyslexia.

**TABLE 3:** Prevalence rates of comorbidity in children with idiopathic epilepsy and schoolchildren under different cutoff criteria (−1 and −1.5 SD)

	Children with idiopathic epilepsy (N = 2282)			Other schoolchildren (N = 2371)		
	% (N)	% of dyscalculia/dyslexia		% (N)	% of dyscalculia/dyslexia	
		+Dyscalculia	+Dyslexia		+Dyscalculia	+Dyslexia
Dyscalculia						
−1 SD	23.2 (530)	/	42.6	10.4 (247)	/	36.4
−1.5 SD	12.3 (281)	/	32.7	5.4 (128)	/	26.6
Dyslexia						
−1 SD	20.8 (474)	47.6	/	12.4 (295)	30.5	/
−1.5 SD	10.5 (240)	38.3	/	6.1 (145)	23.5	/

### 3.2 Gender differences

Gender difference in the prevalence of dyscalculia and dyslexia and their comorbidity are presented in Table 4. For children with idiopathic epilepsy, significant differences between boys and girls were found only in dyslexia under −1 and −1.5 SDs (22.5% vs 17.7%, 11.8% vs 8.3%, respectively, all *P* < 0.01), and isolated dyslexia under −1 SD (11.9% vs 9.1%, χ^2^ = 4.300, *P* < 0.05). No gender differences were found for children with idiopathic epilepsy in dyscalculia or isolated dyscalculia.

On the contrary, there were significant gender differences among other schoolchildren in all categories of learning disabilities under both cutoff criteria. Prevalence rates of dyscalculia and isolated dyscalculia were significantly higher among schoolboys than schoolgirls under both −1 SD (13.1% vs 7.4%, 7.8% vs 5.3%, respectively, all *P*s < 0.05) and −1.5 SD (6.9% vs 3.7%, 4.9% vs 2.9%, respectively, all *P*s < 0.05). Prevalence rates of dyslexia and isolated dyslexia were also significantly higher in schoolboys compared to schoolgirls under −1 SD (16.7% vs 7.6%, 11.5% vs 5.5%, respectively, all *P*s < 0.001) and −1.5 SD (8.4% vs 3.5%, 6.4% vs 2.7%, respectively, all *P*s < 0.001).

**TABLE 4:** Gender difference in the prevalence of dyscalculia and dyslexia and their comorbidity in children with idiopathic epilepsy and other schoolchildren in China

Groups	Children with idiopathic epilepsy		χ^2^	Other schoolchildren		χ^2^
	Boys (N = 1446)	Girls (N = 836)		Boys (N = 1256)	Girls (N = 1115)	
	% (N)	% (N)		% (N)	% (N)	
−1 SD						
Dyscalculia	23.6 (341)	22.6 (189)	.282	13.1 (164)	7.4 (83)	19.943***
Dyslexia	22.5 (326)	17.7 (148)	7.546**	16.7 (210)	7.6 (85)	44.863***
Dyscalculia (Isolated)	12.9 (187)	14.0 (117)	.518	7.8 (98)	5.3 (59)	6.023*
Dyslexia (Isolated)	11.9 (172)	9.1 (76)	4.300*	11.5 (144)	5.5 (61)	26.868***
−1.5 SD						
Dyscalculia	12.4 (180)	12.1 (101)	.066	6.9 (87)	3.7 (41)	12.213***
Dyslexia	11.8 (171)	8.3 (69)	7.183**	8.4 (106)	3.5 (39)	25.122***
Dyscalculia (Isolated)	7.6 (110)	9.4 (79)	2.368	4.9 (62)	2.9 (32)	6.624*
Dyslexia (Isolated)	7.0 (101)	5.6 (47)	1.622	6.4 (81)	2.7 (30)	18.698***

*P < .05; **P < .01; ***P < .001.

## 4 DISCUSSION

The current study examined whether children with epilepsy exhibited a higher prevalence and comorbidity of learning disabilities compared to other schoolchildren as well as gender differences in the prevalence of learning disabilities within two large population‐based samples of children. The results showed that children with epilepsy had significantly higher prevalence rates of dyscalculia, dyslexia, isolated dyscalculia, isolated dyslexia, and comorbidity rates under both −1 and −1.5 SDs cutoff criteria when compared with other schoolchildren. Gender differences were presented in all types of learning disabilities among typically developing schoolchildren, but only in dyslexia among children with idiopathic epilepsy.

The higher prevalence of learning disabilities among children with epilepsy is consistent with prior studies which have shown that different epilepsy syndromes are associated with educational underachievement.^34^, ^35^, ^36^, ^37^ A recent nationwide study in Germany reported higher prevalence of epilepsy in patients with developmental disorders of speech and language compared with those in the general population.^37^ Another recent study reported that childhood epilepsy occurs in a fundamentally abnormal brain that also has a higher risk for neurobehavioral comorbidities, which is conducive to the underdevelopment of academic ability.^38^ In addition, one high risk factor, the epileptic seizure, negatively affects brain development and self‐esteem in children with epilepsy. For example, Cheng et al^39^ reported an association between childhood idiopathic epilepsy with interictal epileptiform discharges and arithmetic performance deficits. Increased frequency of seizures also results in higher vulnerability to disease and low self‐esteem.^40^ Low self‐esteem, in turn, decreases the academic performance in children with epilepsy.^41^

The higher prevalence rate of dyscalculia than dyslexia among children with idiopathic epilepsy could be related to the nature of arithmetic. Comparing to reading tasks, arithmetic tasks require the use of manifold cognitive operations and are therefore more vulnerable to the effects of epilepsy. Specific learning disabilities were shown to be the greatest in arithmetic performance in a study of 122 children with epilepsy.^42^ A study on 94 participants with new‐onset epilepsy and 72 healthy controls found better performance on reading than arithmetic among the epilepsy group and an opposite trend in the control group.^43^ Another study on 136 children who had undergone resective epilepsy surgery found 22% underachieved in arithmetic while only 8%‐9% in other academic domains including reading and spelling.^44^ Other than the complex nature of arithmetic, a lack of direct educational resources in arithmetic compared to reading could further contribute to the more pronounced decrements in arithmetic skills.

An interesting finding of the current study is that children with idiopathic epilepsy also had higher comorbidity for learning disabilities than typically developing children. Specifically, for children with idiopathic epilepsy, the proportion of individuals with dyslexia to those with dyscalculia, and the proportion of individuals with dyscalculia to those with dyslexia, were higher than for typically developing schoolchildren. The overlap between reading and arithmetic deficiencies has been well documented by previous studies.^22^, ^45^, ^46^, ^47^ A proposed explanation for the overlap is that children may develop problems in verbal number tasks if language impairments are present.^47^ Alternative hypotheses for the comorbidity include deficits in verbal short‐term and long‐term memory retrieval.^48^ However, no prior study has examined comorbidity rates of learning disabilities among children with epilepsy or made equivalent comparisons with a control group; therefore, results from the present study could serve as a reference for future examinations on this topic.

Gender differences existed only in dyslexia (−1 and −1.5 SDs) and isolated dyslexia (−1 SD) for children with idiopathic epilepsy, in which boys had higher prevalence rates than girls. A study of 108 probands with rolandic epilepsy (age range: 3.6‐22 years) found that male sex is one of the markers of higher risk for reading disability in children with Rolandic epilepsy.^49^ Nonetheless, most studies on gender differences regarding academic achievement in children with idiopathic epilepsy have found no significant gender effects.^50^, ^51^ Previous systematic investigations on the prevalence of epilepsy in China have reported higher prevalence estimates in males than in females, which could be caused by the inherent differences in brain development and social effects.^52^ The different proportional contribution of epilepsy subtypes could intensify such gender imbalance. Relevant studies have found higher prevalence of localization‐related symptomatic epilepsy in males.^53^ Therefore, the gender imbalance in our epilepsy sample is likely due to that males are more susceptible to epilepsy in general than females. Thus, future studies should examine the effects of other demographic characteristics (such as grades and urban/rural settings) on the prevalence of learning disabilities and should also examine the possible mediation effect of epilepsy‐related clinical variables on gender differences in learning disabilities.

For typically developing schoolchildren, boys exhibited a higher prevalence of dyscalculia, dyslexia, isolated dyscalculia, and isolated dyslexia regardless of cutoff criteria. According to a meta‐analysis on dyslexia, men are more likely to be identified as having dyslexia compared to women, regardless of methodological and statistical influences.^54^ This reading disadvantage in males could be caused by several factors, including (but not limited to) sex differences in cognition^55^ and learning strategies.^56^, ^57^ Moreover, the evidence in typically developing children suggests that girls generally outperform boys in arithmetic.^58^ However, previous studies have reported that gender ratios in arithmetic disorder are usually balanced.^59^, ^60^ Gender differences in the prevalence of dyscalculia in our study could be due to differences in screening criteria used to assess dyscalculia. Dyscalculia has often been defined in terms of performance on arithmetic, in which girls are shown to be more skilled in. Therefore, in the current study, boys had a higher prevalence rate of dyscalculia compared to girls because the test used to define dyscalculia, a simple subtraction test, kept girls’ advantage in arithmetic. These findings altogether indicate that children with epilepsy are at a much higher risk of having learning disabilities when compared to other schoolchildren. Moreover, boys with epilepsy exhibited the highest risk of having dyslexia when compared to all other groups, and typical developing boys were about two times more likely than typical developing girls to have learning disabilities. Our results suggest that pediatric clinicians should pay more attention to the diagnosis and intervention of learning disorders in children with idiopathic epilepsy.

The current study has some limitations. First, our control group was on average 1 year older than the epilepsy group. This is caused by the different sources of data collection. Children with epilepsy from the hospital tend to get diagnosed and treated earlier, therefore most of them were young children. However, schoolchildren were collected from all grades in schools, in which the age had uniform distribution. In an effort to control for the effect of age, the prevalence of learning disabilities was calculated based on standard scores for the arithmetic and reading tests using the mean score and standard deviation for each given age in the sample. Second, we used only arithmetic fluency and reading fluency tests to screen the participants with learning disabilities. These two tests are the typically used assessments, and all participants were capable of performing these tasks. However, it would be better if future research could employ more tests for each domain to assess academic performance. Third, the current study did not examine the effect of syndrome‐related clinical variables, such as type of epilepsy, age at epilepsy onset, epileptic seizure, and treatment. Although syndrome‐related clinical variables have been considered as potential influencing factor of learning disabilities, these factors cannot account for whether learning disabilities occurred or not.^3^, ^21^, ^51^, ^61^, ^62^, ^63^, ^64^ The present study aimed to examine whether children with epilepsy exhibited a higher prevalence and comorbidity of learning disabilities compared to other schoolchildren, rather than focus on the effects of the detailed epilepsy‐related variables on learning disabilities.

## 5 CONCLUSION

This study extends previous findings by examining the prevalence rates of learning disabilities among children with idiopathic epilepsy in China and confirming that they have higher prevalence and comorbidity rates of learning disabilities than other schoolchildren. Typically developing children and children with idiopathic epilepsy had different patterns of prevalence rates for different types of learning disabilities; specifically, typically developing children are more prone to dyslexia than dyscalculia, and the opposite is true for children with idiopathic epilepsy. We also found that there were gender differences in the prevalence of learning disabilities among typical developing schoolchildren, and gender differences in the prevalence of dyslexia among children with idiopathic epilepsy. These findings suggest that children with idiopathic epilepsy have much higher risk to exhibit dyscalculia and dyslexia compared to other schoolchildren, and that boys are generally more at risk than girls to have learning disabilities. Future studies should clarify the effect of clinical variables on the prevalence of learning disabilities in children with idiopathic epilepsy.

## CONFLICT OF INTEREST

None of the authors has any conflict of interest to disclose.

## ETHICAL APPROVAL

We confirm that we have read the Journal's position on issues involved in ethical publication and affirm that this report is consistent with those guidelines.
